# Relationship between Bruises on Carcasses of Beef Cattle and Transport-Related Factors

**DOI:** 10.3390/ani12151997

**Published:** 2022-08-07

**Authors:** Emanuela Zanardi, Silvio De Luca, Giovanni Loris Alborali, Adriana Ianieri, Maria Olga Varrà, Claudia Romeo, Sergio Ghidini

**Affiliations:** 1 Department of Food and Drug, University of Parma, Via del Taglio 10, 43126 Parma, Italy; 2 Istituto Zooprofilattico Sperimentale della Lombardia e dell’Emilia Romagna-Headquarters, Via A. Bianchi, 9, 25124 Brescia, Italy

**Keywords:** cattle welfare, beef, bruises, post-mortem, space allowance, transport

## Abstract

**Simple Summary:**

The use of monitoring schemes to determine the health and welfare status of animals at the abattoir level provides useful feedbacks to farmers and to all those involved in the activities carried out before slaughtering. Bruises are considered welfare indicators in cattle carcasses, as they represent a sign of improper handling or management executed during the pre-slaughter phases. The aims of this study were to assess the prevalence of bruises in beef cattle at the slaughterhouse and to determine their correlation with factors linked to animal transport. The 21.6% of the animals included in the study had bruises, which were mainly characterized by being red, small and located in one region. Steers had less probability of presenting bruises compared to the other categories, while animals transported in the condition of a high space allowance had more chance of presenting bruises. Even though more studies are needed, the results of this study have shown that the assessment of bruises on cattle carcasses could reflect some welfare issues occurring during pre-slaughter activities. These data can be used to improve specific procedures and to boost cattle welfare.

**Abstract:**

The assessment of bruises on carcasses at the slaughterhouse has been lately indicated as a valid method to evaluate cattle welfare. However, little is known about the prevalence and the causes of bruises of cattle slaughtered in Italy. The aim of this study was to collect information concerning the prevalence of bruises on the carcasses of beef cattle slaughtered in an Italian abattoir and to determine a relationship between fresh bruises and transport-related factors. In total, 1265 animals were included in this study, with 21.6% of them being positive for at least one bruise, either fresh or old. In most cases, the bruising was mild, with lesions exclusively located in one area of the carcass. Most of the bruised animals (63%) showed at least one red lesion. Occurrence of such red, fresh bruises varied significantly depending on the body parts (*p* < 0.0001), with the flank being the most affected area (39.5%), followed by the butt (36.0%) and the front (23.8%). The probability of fresh bruising varied significantly depending on the category of each animal (*p* < 0.0001), with steers showing fewer red bruises than both heifers and veal. Finally, animals transported in conditions of a high density had a lower probability of bruising (*p* = 0.0003). These findings support the use of a monitoring scheme based on the presence of bruises to assess cattle welfare at the abattoir level in order to provide feedback to farmers and to implement procedures carried out during transport.

## 1. Introduction

The assessment of animal welfare at the slaughterhouse is a relatively recent topic that has been raising the interest of the scientific community in recent years [1,2]. In fact, at the abattoir level it has become possible to determine the status of the welfare of animals through the analysis of specific animal-based indicators located on the carcasses of the animals [3]. In regard to this, a large number of references are available when it comes to the determination and validation of animal-based welfare schemes at the slaughterhouse for certain species, such as pigs and poultry [4,5,6,7,8], while, on the other hand, little is known about the reliability of specific indicators at the abattoir level to determine welfare in cattle.

Recently, the evaluation of bruises on carcasses has been advocated as a possible monitoring system to assess the welfare in cattle at the slaughterhouse, considering the fact that the presence of these lesions has been related to some pre-slaughter procedures such as transport, the overcrowding of lorries or rough handling during the unloading or stunning [9]. Bruises are caused by the rupture of blood vessels that lead to blood accumulation in the muscle and other tissues without evident damage of the skin, resulting from the impact coming from the surrounding environment, a conspecific or due to human–animal interactions [10]. In cattle, the presence of different types of hair coats and the thickness of the skin make these lesions visible as distinct discolorations on carcasses only after the removal of the skin step (“skinning”) at the slaughterhouse [10,11]. Apart from the welfare assessment prospect, the presence of bruises can affect producers from an economical and technological point of view, as bruised areas on carcasses require separated trimming, leading to a reduced yield and a possible decrease in the carcass value and, depending on the bruise location, a potential devaluing of cuts [11]. Moreover, Romero et al. [12] have shown that the pH of carcasses presenting bruises was likely to be higher than 5.8, which is the value of pH related to dark, dry and firm meat (DFD) meat.

Most of the scientific literature currently available includes data from studies performed on cattle slaughtered in South America [11,12], while little is known when it comes to information regarding cattle slaughtered in Europe, even though some differences are present between these two continents. For example, beef cattle production systems in South American countries are predominantly based on breeding on large pastures, while feedlots systems are more commonly employed in European cattle farms [13]. Moreover, the European legislation concerning animal welfare is generally considered to be more stringent with regard to the requirements to be fulfilled to maintain adequate animal welfare during transport or at the slaughterhouse compared to South American countries [14]. With regard to this, Article 21 of the Regulation 625/2017 on the official controls and other official activities carried out by the European competent authorities on feed and food of an animal origin specifically advocates for the adoption of scientifically validated indicators for the assessment of animal welfare along any point of the food chain, thus including welfare outcomes to be evaluated at the abattoir level [15]. Furthermore, very little is known on the presence of bruises on cattle raised and slaughtered in Italy [16], where the production of beef cattle is economically relevant and strictly connected with rural and ethnic traditions [17]. 

The aim of this study was, therefore, to evaluate the presence and the distribution of bruises on beef cattle and veal carcasses and to assess the role of some potential transport-related pre-slaughters risk factors. 

## 2. Materials and Methods 

### 2.1. Abattoir Layout and Handling Operations 

The study was conducted on 1265 cattle slaughtered in an abattoir located in northern Italy, in the province of Padua (PD), during an observational two-month period from May to June 2021, for a total of 12 visits. This slaughterhouse processed mainly beef cattle, culled cows and white veal and it operated according to a schedule of four working days per week, with the first animal stunned each day at around 6:30 am. In regard to this study, the examined animals were divided in the following commercial categories: 1—veal, male or female under 12 months of age; 2—steers, castrated males between 1 and 3 years of life; 3—heifers, females between 1 and 3 years of age [12]. Due to the low occurrence of culled cows during the time of the study, these were not included in the study. All the animals comprehended in our investigation were de-horned. 

Overall, the plant had a processing capacity of about 120 animals per day at a rate of 30 animals per hour. Most of the animals processed in this abattoir were collected from farms located in the vicinity of the slaughterhouse (i.e., approximately within a 100 km radius), but the plant also received animals from farms located in other provinces of the same regional area (Region of Veneto, Italy). As per Regulation (EU) 1760/2000 [18], each animal was identified by a unique number recorded on a passport held by the keeper since the animal’s birth and kept throughout the entirety of its life. Once at the slaughterhouse, after checking the passports, cattle and veal were moved from the transportation lorries to the lairage pens through a concrete unloading ramp with nonslip floors by a single operator with the help of a stick when needed. Each pen had a solid concrete floor without bedding, with water freely available to the animals, but with no access to feed. It must be noted that animals from different livestock trucks were not mixed when placed in the lairage pens. Moreover, the lairage time of the animals included in this study was never longer than 60 min and was, therefore, not included among the possible factors affecting the presence of bruises on cattle carcasses. When required, the animals were conveyed from the lairage to the race and then sorted in a single-line into a straight corridor, which led to the stunning box. The operators did not use electric prods during the movement of the animals from the race to stunning box, but sticks and shouting were used as driving instruments. Here, the animals were stunned using a captive bolt pistol, hung by their hind legs and exsanguinated by severing the main vessels located in the neck area, as required by the Council Regulation (EC) 1099/2009 on the protection of the animals at the time of killing [19]. Following this step, the cattle were introduced in the slaughter hall and further processed along the slaughter line.

### 2.2. Pre-Slaughter Factors 

All the animals included in this study were transported into livestock trucks complying with Regulation (EU) 1/2005 for the protection of animals during transport at the slaughterhouse [20]. The examined animals were unloaded from a total of 65 truckloads transported by 13 different drivers (median no. of loads per driver = 2; range= 1–15). The majority of drivers carried out deliveries for multiple farms and often through different types of trucks. Data concerning transport-related factors were collected through a survey directly administered to each truck driver or through the examination of the Food Chain Information (FCI) documents. All the data were collected following the unloading of the animals from each livestock truck. The collected pre-slaughter factors were: (I) the name (ID) of the driver, (II) the number of truck floors, (III) the truck type, (IV) the truck size (space available for the animals), (V) the transport duration (in minutes) and (VI) the number of transported animals.

### 2.3. Bruise Scoring

Bruises on carcasses were scored at the slaughter line, prior to the splitting step, with the head still attached to the body and the entire carcass hanging by both hind-legs. Each carcass was identified after skinning through the assignment of a sequential number within each load. 

**Figure 1:**
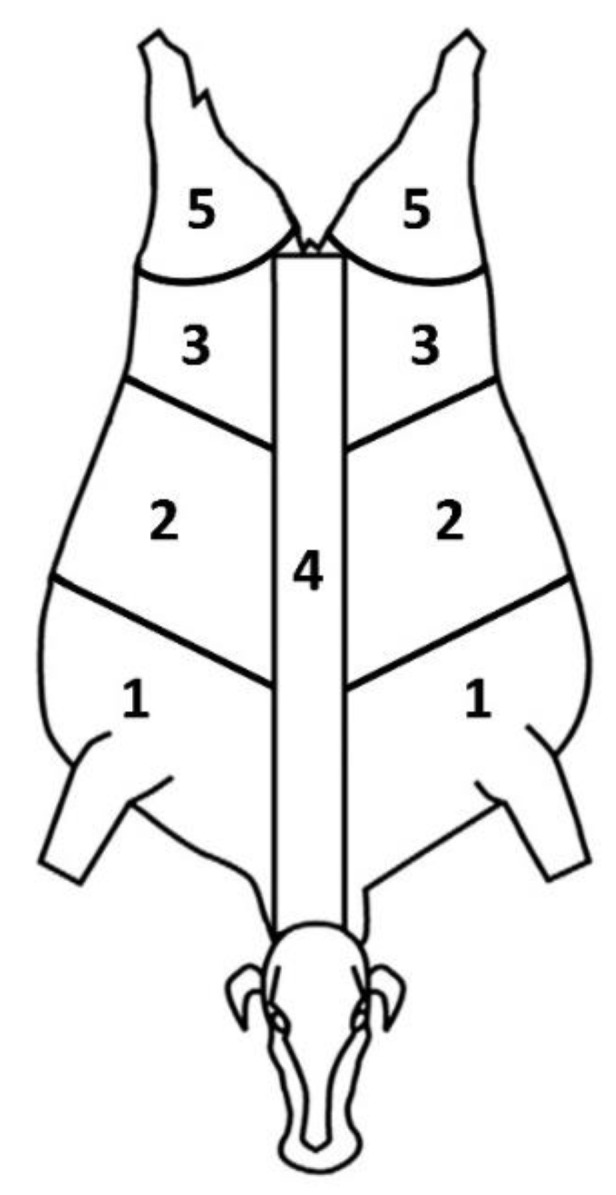
Carcass anatomical sites used for bruises scoring: 1 = Front, 2 = Rib, 3 = Flank, 4 = Loin, 5 = Butt (Adapted with permission from [12], 2013, Elsevier Ltd.).

Each carcass was first checked for the presence of any bruising. In the presence of lesions, the number of bruises per each site was annotated. In order to define the location of the bruises, the carcasses were schematically divided into five different anatomical sites (Figure 1). 

The lesions were further classified according to a protocol adapted from Romero et al. [12] and Knoch et Carroll [20], as detailed in Table 1. 

With regard to bruises shape and size, when a bruise was found to be not circular, its size was measured as the longest length of the lesion [12]. Moreover, when multiple bruises were found on the same site, only the bruise presenting the highest score of the variable ‘Size’ was recorded [20]. 

### 2.4. Statistical Analysis

Firstly, we examined the occurrence of overall bruising detected on cattle at the slaughterhouse, including both old and fresh bruises. The prevalence, body location, characteristics (i.e., color, shape and size) and severity of bruises in our sample are described. Regarding severity, to obtain a single bruising score per animal, for each bruising location (front, flank, rib, loin, butt) the bruise size (1 = small, 2 = medium and 3 = large) was multiplied for the number of bruises of that size. All the scores at different locations were then summed. Finally, additional points (1–5) were added based on the number of body parts of that animal showing at least one bruise. The obtained scores ranged from 2 to 22 and were then split into three categories: mild bruising (score < 6), medium bruising (score between 6 and 10) and severe bruising (score > 10).

Secondly, we focused exclusively on red-colored bruises, i.e., fresher bruises that had likely occurred during transportation, excluding both yellow and purple bruises that could have occurred at an earlier stage on the farm. 

The probability of fresh bruising was examined through a mixed logistic regression, using presence/absence of red-colored bruises on an animal as the response variable (1/0). Individual categories (heifers, steers or veal), transport duration (min), density of animals in the truck (no. of animals/m^2^), no. of floors in the truck (1 or 2), truck size (small: <17.5 m^2^, medium: 32–37 m^2^, large: 68 m^2^) and type of truck (single-unit 3-axles truck or single-trailer 5-axles truck) were included as explanatory variables. The driver ID was included as a random intercept term to account for driving differences. The severity of fresh bruises was examined through a second model by including only the subset of animals showing at least one red bruise (n = 172). The three categories (mild, medium and severe) derived from the bruising score re-calculated excluding older bruises, were used as response variable in a mixed ordinal logistic regression, including the same variables of the first model as explanatory variables and driver IDs as random intercepts.

Differences between factors with more than two levels were tested through t-tests on differences of least square means, applying Tukey adjustment for multiple comparisons. Significance of the covariance parameter was tested by comparing the mixed model and a null model without random terms through a likelihood ratio test. Unless otherwise indicated, values are reported as mean ± Standard Error (SE). All the analyses were carried out using SAS/STAT 9.4 software (Copyright © 2011, SAS Institute Inc., Cary, NC, USA).

## 3. Results

Overall, 1265 animals were examined for bruises post-slaughter, with 273 animals (21.6%; 95% Confidence Interval: 19.3–23.8%) showing at least one bruise. A detailed breakdown of bruises’ prevalence by individual categories and truck characteristics is reported in Table 2. The mean transport duration was 106 ± 2 min (range: 10–1440), while the mean density of animals in a truck was 0.53 ± 0.004 animals/m^2^ (range: 0.01–0.83).

**Figure 2:**
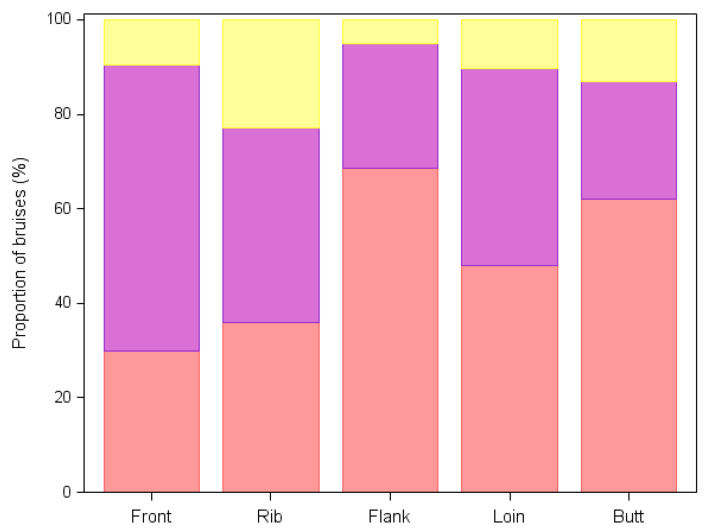
Proportion of fresh (red), old (purple) and very old (yellow) bruises in the different body parts of bruised bovines (n = 273) examined at the slaughterhouse.

Taking into consideration only the 273 animals showing at least one bruise, 193 (70.7%) showed mild bruising, 59 (21.6%) showed medium bruising and 21 (7.7%) showed severe bruising. The median number of bruises per animal was two (range: 1–8). Most of the bruised animals (50.2%) showed bruising at a single location on their body, 38.5% at two locations, 9.2% at three locations and only six individuals (2.2%) showed bruising at four locations. The occurrence of bruising varied significantly depending on the body parts (χ^2^_4_ = 83.2; *p* < 0.0001): 50.2% of bruised animals showed at least one bruise on their front, followed by bruises on the butt (36.6%) and flanks (36.3%), while only 22.3% and 17.6% of the animals showed bruises on their ribs and loins, respectively (Figure 2).

**Figure 3:**
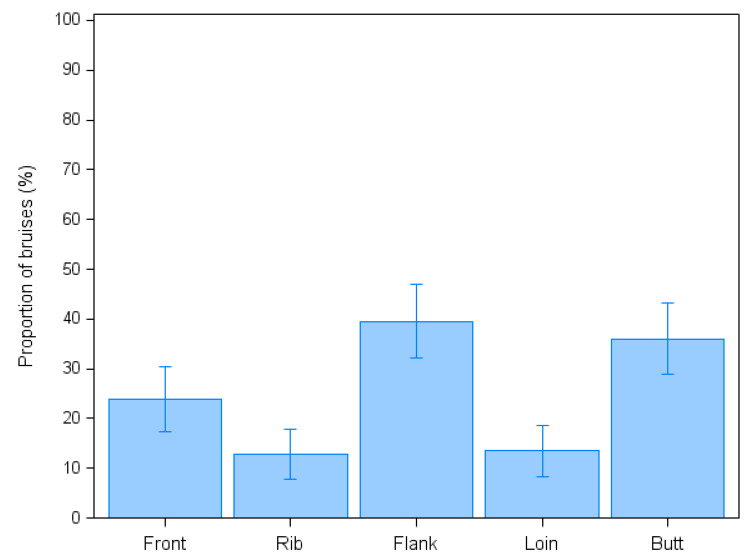
Occurrence of bruising by body part in freshly-bruised bovines (n = 172) examined at the slaughterhouse. Error bars represent 95% Confidence Interval.

Overall, most of the observed bruises were irregular in shape (42.2%), followed by circular (29.2%) and linear (27.4%). Only a few “tramline” bruises were observed (1.1%). With respect to color, most of the observed bruises were fresh lesions (red, 48.5%), followed by old lesions (purple, 40.2%) and very old lesions (yellow, 11.2%). However, this general pattern varied depending on the body part: for instance, most of the bruises observed in the front were purple in color rather than red (Figure 3). 

Focusing on fresh, potentially transport-related bruising, 172 out of 1265 examined animals (13.6%; 95% CI: 11.7–15.5%) showed at least one red bruise, corresponding to the 63.0% of the 273 bruised bovines. The median number of red bruises per animal was one (range 1–8) for a total of 268 examined lesions. Most of these fresh lesions were small or medium-sized (57.4% and 28.2%, respectively) and of a circular (40.3%), irregular (31.0%) or linear shape (27.8%). Regarding their location, most of the freshly-bruised animals showed at least one red lesion on their flanks (39.5%), butt (36.0%) or front part (23.8%), while fresh lesions on ribs and loins (12.8% and 13.7%, respectively) were significantly less common (χ^2^_4_ = 56.6; *p* < 0.0001; Figure 3). Finally, the median individual score for fresh bruises was three, ranging from two to fourteen. The vast majority of freshly-bruised animals (86.6%) showed only mild bruising, 9.9% medium bruising and only 3.5% severe bruising.

The probability for fresh, transport-related bruising varied significantly among individual categories (χ^2^_2_ = 35.4; *p* < 0.0001) and was negatively affected by the density of animals in the truck (χ^2^_1_ = 13.2; *p* = 0.0003). In detail, steers had a lower probability of showing bruises than both heifers (t_1244_ = 5.7; *p*_adj_ < 0.0001) and veal (t_1244_ = 3.7; *p*_adj_ = 0.0006), while the higher the density of transported animals, the lower the probability of bruising (parameter estimate= −2.30 ± 0.63). All the other examined variables had no effect on the presence/absence of fresh bruises (all *p* > 0.05) and the driver was not a source of variation since its inclusion did not improve the fit of the model (χ^2^_1_ = 0.11; *p* = 0.37). Regarding variation in the severity of fresh bruises, it was not affected by any of the examined variables (all *p* > 0.05). 

## 4. Discussion

In recent years, abattoir-based welfare schemes are being more and more frequently adopted in European slaughterhouses in order to assess the welfare and health status in a number of species, such as sheep [21], poultry [22] and pigs [23,24]. However, only few studies have been carried out so far when it comes to cattle slaughtered in Europe. In this study, a scheme based on the presence and severity of bruises on carcasses of beef cattle and veal slaughtered in an Italian abattoir was used and the correlation between bruises and some transport-related factors was explored. Bruises are the consequence of traumatic events, which can occur at any point of the different stages on the day of slaughter [25]. Therefore, their assessment on cattle carcasses may be indicative of the different issues that occur during any steps preceding the slaughter. In our study, the overall prevalence of animals presenting at least one bruise was 21.6%, which is similar to the prevalence of bruises reported by Strappini et al. [26] in Chile, Betancourt-Garcia et al. [27] in Brazil, and slightly lower than the prevalence outlined by Romero et al. [12] in Colombia. On the other hand, our prevalence was different from the prevalence reported in other studies, in which the percentage of animals reporting bruises was even more that the 90% [20,28]. Such a disagreement among studies could be explained by a number of factors, such as the differences in terms of transport duration and distance (from either farms or markets) and the cattle functional type (beef or dairy) included in the different datasets. Mendonça et al. [29] have reported that cattle transported along distances greater than 240 km resulted with more bruises in the hip region compared to cattle transported for shorter journeys. Similarly, Bethancourt-Garcia et al. [27] reported that the percentage of bruises on cattle carcasses was related to the distance traveled on unpaved roads, with animals transported from more than 30 km presenting more bruises compared to animals transported for less than 30 km. In our study, most of the animals were transported from farms in the proximity of the slaughterhouse, with a mean transport duration of 106 ± 2 min. This could partially justify the low prevalence of bruises that has been found in the present study compared to others. 

Taking into consideration all the bruises, our results show that most of the animals had mild bruising, with the majority of recorded bruises characterized by being small, red and found at a single location, in agreement with the results of previous studies [11,30]. Notably, some anatomical sites, such as the front and the ribs of the carcasses showing at least one bruise, presented a relatively high percentage of old (violet) and very old (yellow) bruises compared to other anatomical sites. Such lesions could have been consequential to traumatic events occurred prior to the transport, for instance, during the gathering and mixing of animals that is normally carried out at the farm level the day before transport [31]. 

Fresh bruises were conversely mainly located on the flanks and the butt of the animals, with the loin resulting as the least affected area, in contrast with previous investigations. In fact, the loin area has been often indicated as one of the most frequent location of bruises [9,32]. In particular, this site has been previously related to improper handling carried out at the abattoir level, as highlighted by Strappini et al. [30], who suggested that the main cause of bruising on the loin was the impact with a metallic gate controlled by the operators when an animal was entering the stunning box, thus indicating a possible effect of inappropriate maneuvers performed by the operators. Other potential causes of fresh bruises on this area are the mounting and agonistic behaviors among cattle that can be triggered during the time spent in the lairage, as the animals find themselves in a new environment or mixed with unfamiliar individuals [33]. Agonistic behavior during lairage could also cause the presence of bruises in other anatomical regions, such as the neck, the flank and the hindquarters, considering that during their fights, the animals can hit surrounding structures and objects [34]. In our study, the lairage time was of short duration (less than 60 min): this element could help explaining the low prevalence of lesions of the loin compared to other studies and could also imply that the fresh lesions detected in this study occurred mostly during the loading, transport or unloading steps. As per the other anatomical sites, the presence of bruises on the flank or butt area, which are the two most affected anatomical regions in our study, could be induced by rough handling with driving instruments, such as wooden sticks, performed by the operators when they want to quickly move a reluctant animal during the loading step. In fact, in a study conducted by Strappini et al. [30], 23.1% of the bruises were caused by rough handling during ] loading and all these lesions were located on the pin site, which is included in the flank area in the assessment scheme used in our study. Moreover, the improper use of driving instruments could also enhance the excitement of the animals during the loading step, which can result in the animals hitting the truck walls or losing their balance, increasing the chance of bruises in other regions [34]. Even though some bruises can be linked to specific traumatic events, it should be noted that this is not always the case. As an example, Lee et al. [32], in a study aimed at linking specific bruises with hurtful episodes during unloading (e.g., hitting any part of a trailer) at the slaughterhouse, have not found a direct correlation between these factors. Similarly, Jarvis et al. [35] were not able to observe a direct relation between traumatic events at the abattoir and bruises. As previously stated, the occurrence of bruising on cattle carcasses depends indeed on several elements. In this context, handling procedures may of course play a critical role, but other factors such as the gender, the animal types and the loading density during transport need to be taken into account as well. 

As a matter of fact, our results have shown that steers had a significantly lower probability of presenting fresh bruises compared to both heifers and veal. In regard to this, the role played by both sex and age has been previously highlighted in other studies [20]. Female cattle tend indeed to present more bruising compared to male cattle due to the fact that they may express a higher degree of aggressiveness, which may result in problematic handling and higher chances of collisions with the different facility structures along the steps prior to slaughter [36]. Moreover, female cattle are generally characterized by a lower percentage of muscle tissue compared to adult male cattle: these are two important factors that have been previously related to an increased occurrence and severity of bruising [25,29]. Similarly, young cattle may have a lower fat percentage compared to steers, which would make this category of animals more susceptible to bruising [20]. On the other hand, Romero et al. [12] reported a greater occurrence of bruising in males compared to females (41.2% vs. 26.5%). Such differences may be representative of the different animal types involved in the different studies, highlighting the importance of a multifactorial and integrated approach when evaluating risk factors for bruises in cattle, as outlined by Kline et al. [9]. Interestingly, in this study, the probability of having fresh bruises was negatively affected by the density of animals in the truck, which means that the occurrence of bruises in animals transported in conditions of a high load density was lower compared to animals transported in low density conditions. Regarding this topic, there are some inconsistencies in the literature currently available. A high load density during transport has been previously associated with an increased occurrence [12,29,37] or greater severity [27] of bruises. In fact, higher densities can lead to enhanced stress responses and to an increased risk of falling or losing balance [37]. On the other hand, other authors have reported that animals transported in conditions of low densities are at risk of slipping or losing balance due to the presence of the extra space allowance [38]. In the study by Eldridge and Winfield [39], the bruises score recorded from carcasses of beef cattle transported in a medium space allowance (1.16 m^2^ per animal) was significantly lower compared to beef cattle transported in a low (0.87 m^2^ per animal) and high space allowances (1.39 m^2^ per animal). Similar findings were also reported by Mendonça et al. [40], with animals transported in a moderate density condition presenting fewer bruises than animals transported in either low or high density conditions. In this view, further research on the effect of the space allowance on cattle bruising should be carried out in order to better understand the impact of this crucial factor on cattle welfare. 

## 5. Conclusions

The presence of bruises on cattle carcasses represents a great concern in the beef industry given their negative impact on animal welfare and on the commercial values of carcasses. The results of this study have shown that the distribution and the severity of bruises may be affected by some pre-slaughter factors, such as the animal category and the load density, although other variables concerning the procedures carried out at the farm level should be considered for further studies. The application of a welfare scheme to determine the distribution and severity of bruises on cattle could be useful for both producers and competent authorities to characterize cattle farms, transporters and slaughterhouses following a risk-based approach. These schemes could be also integrated into standard meat inspection procedures which are normally carried out at the abattoir level, reducing the need for on-farm visits. The data acquired through the application of an abattoir-based welfare scheme could further be used to implement or to correct procedures carried out during the pre-slaughter activities or to improve the design of the areas dedicated to such procedures, reducing the need for driving instruments and generally improving cattle welfare. 

## Figures and Tables

**Table 1:** Bruises classification adopted in this study.

Variables	Description
Size	Small: 1–8 cm Medium: 9–16 cm Large: >16 cm
Shape	Circular: a bruise in the shape of a circle Linear: a bruise with one dimension (length) longer than the other (width) Tramline: two parallel linear bruises separated by a paler undamaged area Irregular: a bruise without clear dimensions and with uneven edges
Color	Red: fresh lesion Purple: old lesion Yellow: very old lesion

**Table 2:** Prevalence (%) of bruising observed post-slaughter in bovines (n = 1265) by individual category and transport characteristics.

		N	% of Bruised Animals	95% CI
Individual category	Veal	89	29.2	19.6–38.8
	Steers	636	15.1	12.3–17.9
	Heifer	540	28.0	24.2–31.8
Truck size	Small	160	18.7	12.6–24.9
	Medium	94	24.5	15.6–33.3
	Large	1011	21.8	19.2–24.3
Truck type	Single-unit 3-axles	271	22.5	17.5–27.5
	Single-trailer 5-axles truck	994	21.3	18.8–23.9
No. of floors	1	165	19.4	13.3–25.5
	2	1100	21.9	19.5–24.4

## Data Availability

The data included in this study can be provided upon direct request to the corresponding author.
